# Dynamics of invasive pneumococcal disease in infants < 2 years old following PCV7/13 implementation using two infant and a booster dose schedule: evidence for indirect protection of young infants, Israel, 2004 to 2019

**DOI:** 10.2807/1560-7917.ES.2023.28.25.2200765

**Published:** 2023-06-22

**Authors:** Shalom Ben-Shimol, Bart Adriaan van der Beek, Meirav Mor, Orli Megged, Ron Dagan, on behalf of the Israeli Pediatric Bacteremia and Meningitis Group (IPBMG) and the Microbiology Group

**Affiliations:** 1 Faculty of Health Sciences, Ben-Gurion University of the Negev, Beer Sheva, Israel; 2 Pediatric Infectious Disease Unit, Soroka University Medical Center, Beer Sheva, Israel; 3 The Shraga Segal Dept. of Microbiology, Immunology, and Genetics, Faculty of Health Sciences, Ben Gurion University of the Negev, Beer Sheva, Israel; 4 Infectious Diseases, Schneider Children's Medical Center of Israel, Petah Tikvah, Israel; 5 Pediatric Infectious Diseases, Shaare Zedek Medical Center, Jerusalem, Israel and Faculty of Medicine, Hebrew University, Jerusalem, Israel; 6 The members of the Israeli Pediatric Bacteremia and Meningitis Group (IPBMG) and the Microbiology Group have been listed at the end of the article

**Keywords:** Herd protection, indirect protection, IPD, 2+1 vs 3+1 schedule, PCV13, infants

## Abstract

**Background:**

Pneumococcal conjugated vaccine (PCV)7 and PCV13 programmes started in Israel from July 2009 and November 2010 respectively, with a 2+1 schedule (one dose at 2 months old, one at 4 months old, and a booster dose at 12 months old). Thereafter, invasive pneumococcal disease (IPD) rates substantially declined in children. Uptake of all three doses in < 2-year-olds since 2012 is > 90%. For still incompletely vaccinated infants (≤ 12 months old), how well the PCV 2+1 programme shields from IPD is not fully resolved.

**Aim:**

To assess the adequacy of protection conferred by the 2+1 schedule PCV vaccination programme, particularly among incompletely-vaccinated infants.

**Methods:**

This was a population-based, prospective, nationwide active IPD surveillance study in Israel, 2004–2019, in children < 24 months old. We estimated annual incidence rates (IR) of overall IPD, IPD caused by PCV13 serotypes (VT13), and non-PCV13 serotypes (NVT13). Annual IPD IRs were stratified by age: < 4 months (receiving ≤ 1 dose), 4–6 months (immediately post dose 2), 7–12 months (a few months post dose 2), and 13–23 months (post dose 3). Late-PCV (2004–2008) to pre-PCV13 (2016–2019) mean annual IR ratios (IRRs) were calculated.

**Results:**

2,569 IPD episodes were recorded. VT13 decreased > 90% in all age groups, while NVT13 seemed to increase. All-IPD rates declined in all age groups by 56–70%. The 2+1 schedule impact on 7–12-month-old infants (pre-booster) was similar to that on 13–23-month-old children (post booster), with PCV13 IPD reductions of 97% and 98%, respectively.

**Conclusions:**

Indirect (herd) protection of infants, including < 4 month-olds with ≤ 1 PCV dose, was achieved by the 2+1 PCV schedule programme which thus seems adequate.

**Key public health message**

**What did you want to address in this study?**

To prevent invasive pneumococcal disease (IPD) in Israel, pneumococcal conjugated vaccines (PCV) are administered in a 2+1 schedule: two successive vaccine doses are given in infancy, then a booster dose at 12 months old. Vaccinating children somewhat shields other population groups vulnerable to IPD, from IPD. We wondered if, as did the originally licensed 3+1 schedule, a 2+1 PCV schedule adequately protects incompletely-vaccinated infants.

**What have we learnt from this study?**

The PCV programme in Israel impacted the occurrence of IPD in several age groups of children. The impact of PCV13-serotype IPD among 7–12-month-old infants (receiving only 2 doses) was similar to that among 13–23-month-old children, receiving also the booster dose, with 97% and 98% reductions of IPD in these two groups, respectively. Similar reductions (> 90%) were also observed in < 7-month-old infants.

**What are the implications of your findings for public health?**

Our results strongly support the 2+1 PCV regimen in Israel, which is also prevalent in Europe, and show that in a country with high vaccination coverage, a third infant dose prior to the booster (3+1 regimen) is not needed.

## Introduction

The implementation of pneumococcal conjugated vaccines (PCVs) into paediatric national immunisation programmes (NIPs) has resulted in a substantial decline of invasive pneumococcal disease (IPD) rates in children [1–4]. Additionally, impact on IPD rates in adults (through indirect/herd protection) has been observed [5,6], with a net decrease of IPD rates in adults [7–9]. Nevertheless, questions have been raised regarding the extent of indirect (herd) protection from vaccinated children to non-vaccinated or partially-vaccinated infants, receiving ≤ 2 PCV doses.

Several factors may affect indirect PCV impact on young infants, including the number of polysaccharide antigens included in the vaccine, the vaccine uptake, the vaccine serotypes, and the locally circulating non-vaccine serotypes, as well as living conditions (e.g. crowding) [5,10,11].

A major factor that should be considered, when assessing herd protection for young infants, is the PCV schedule used. Initially, in the United States, the 7-valent and the 13-valent PCVs (PCV7, PCV13) were licensed and implemented in a 3+1 schedule, with PCV doses given at the ages of 2, 4, 6 months and a booster at 12–15 months. Subsequently, most countries implemented a 2+1 schedule (2 doses in early infancy and a booster dose in the second year of life), while others used a three-dose primary series with no booster dose (3 + 0 schedule) [12,13]. Recent studies suggested that the immunogenicity of the PCV booster dose using only one dose during early infancy (1+1 schedule) resulted in similar immunogenicity to that of the 2+1 schedule [10,11,14]. The main advantages of the reduced doses’ schedules include lower cost, as well as reduced complexity and burden of the vaccination schedule. Nevertheless, reduced schedules bear the risk of incomplete protection for young infants, especially during the period between receiving the priming and the booster PCV doses.

PCV7 was licensed in Israel in 2007 and was introduced to the private market in mid-2008 with variable use. In July 2009, PCV7 was introduced to the Israeli NIP (2+1 schedule; 2, 4, and 12 months) with a catch-up campaign in children younger than 2 years. In November 2010, PCV13 replaced PCV7, without further catch-up [1,2]. The vaccine uptake of all three doses exceeds 90% since 2012. We have previously reported that within 3 years from PCV7/PCV13 implementation, IPD caused by PCV13 serotypes (VT13) rapidly declined by > 90%, along with an increase in IPD caused by non PCV13 vaccine serotypes (NVT13), resulting in an approximately 65% reduction in overall IPD rates in children < 5 years old [2].

We assessed annual IPD incidence dynamics in children < 24 months of age between 2004 and 2019, and compared IPD rates in late-PCV13 (2016–2019) period vs pre-PCV (2004–2008) period by age groups: < 4 months (receiving ≤ 1 dose), 4–6 months (expected to be shortly after dose 2), 7–12 months (several months post dose 2), and 13–23 months (post dose 3 (booster dose)).

## Methods

This was a population-based, prospective, nationwide active surveillance of IPD in Israel, 2004–2019. 

### Setting and study population

For incidence rate estimates, we used the population numbers by age group as the surveillance catchment numbers. There were 286,500, 311,500 and 367,300 children < 24 months of age in Israel in the years 2004, 2009, and 2019, respectively [15].

The study was conducted by the Israeli Pediatric Bacteremia and Meningitis Group (IPBMG) and the Microbiology Group in 27 medical health centres routinely obtaining cerebrospinal fluid (CSF) and blood cultures from children [1,2]. The detailed descriptions of data assembling were previously published [1,2], including vaccine uptake, bacteriology, and microbiological data. We covered > 99% of all culture-confirmed blood and CSF paediatric IPD cases in Israel. Completed reports included the following data: isolate source (blood/CSF), culture date, birth date, sex (male/female), ethnicity (Jewish/non-Jewish), main diagnoses, outcome (mortality), and hospitalisation duration. Laboratory methods were described in detail previously [1].

### Case definitions

All definitions were used as previously described [16]. An IPD episode was defined as an illness episode during which *Streptococcus pneumoniae* was isolated from blood, CSF, or both. Serotyping was performed by the Pediatric Infectious Disease Unit laboratory at the Soroka University Medical Center, Beer Sheva, using a Quellung reaction (Staten Serum Institute, Copenhagen, Denmark).

Non-culture diagnoses (PCR, antigen testing, Gram stain results, or clinical diagnosis) were excluded. Positive cultures from sterile sites other than blood or CSF (i.e. pleural, synovial, or ascitic fluid) were also excluded.

Local investigators in each centre reported the specific IPD clinical syndrome, including meningitis, bacteraemic pneumonia, other IPD sources (bacteraemia with mastoiditis, etc.), and bacteraemia only (without apparent focus).

### Vaccine uptake

Vaccine uptake evaluation methods were previously described [1,2]. By June 2011 and December 2012, approximately 80% and 90%, respectively, of 7- to 11-month-old children received two or more PCV7 and/or PCV13 doses, and approximately 95% received two or more PCV13 doses by June 2014.

### Data analysis

IPD was analysed as follows: (i) IPD caused by VT13, (ii) IPD caused by NVT13, and (iii) overall IPD.

For comparison purposes, in addition to continuous graphs and tables, we defined the following periods (each study year from July to June): pre-PCV (2004–2008), and late-PCV13 (2016–2019). We did not include the year 2008/09 in the pre-PCV years since PCV7 had already been introduced into the private market during this year.

Annual incidence rates were calculated as previously described [16] for the following age groups: < 4 months (receiving ≤ 1 dose), 4–6 months (expected to be shortly after dose 2), 7–12 months (several months post dose 2), and 13–23 months (post dose 3, which is the booster dose).

For pre-PCV episodes in which serotypes were missing, extrapolation was conducted, as previously described [1,2]. However, for post-PCV episodes > 95% of the isolates were serotyped. Data were analysed using SPSS 26.00 software (IBM Corporation, Armonk, New York).

Incidence rate ratios (IRRs) and their 95% confidence intervals (95% CI) were calculated by dividing the mean annual incidence rates during the late PCV period by those during the pre-PCV period.

## Results

Overall, 2,569 IPD episodes were recorded. Of these, 392 (15.3%), 212 (8.3%), 768 (29.9%), and 1,197 (46.6%) were in children < 4, 4–6, 7–12, and 13–23 months of age, respectively.

### Dynamics of invasive pneumococcal disease caused by vaccine serotypes

In the pre-PCV period, the annual mean VT13 IPD incidence rates per 100,000 ± standard deviation (SD) were stable and were 20.8 ± 7.9 and 12.3 ± 1.5 in children < 4 and 4–6 months old, respectively, and 49.4 ± 2.9 and 82.7 ± 4.8 in children 7–12 and 13–23 months old, respectively (Table).

**Table:** Annual incidence of PCV13 serotypes, non-PCV13 serotypes, and overall IPD per 100,000 individuals aged < 4, 4–6, 7–12, and 13–23 months old, Israel, July 2004–June 2019 (n = 2,569 IPD episodes)

Period	< 4 month-olds			4–6 month-olds			7–12 month-olds			13–23 month-olds			
	VT13	NVT13	Total	VT13	NVT13	Total	VT13	NVT13	Total	VT13	NVT13	Total
2004/05	16.01	3.48	19.49	11.83	2.09	13.92	50.12	11.83	61.96	81.31	6.25	87.56
2005/06	32.54	0.00	32.54	12.46	2.08	13.85	45.00	9.00	54.00	89.81	3.45	93.26
2006/07	18.32	8.82	27.15	14.25	3.39	17.65	51.58	6.79	58.36	79.06	8.25	87.32
2007/08	16.51	1.32	17.83	10.56	2.64	13.21	50.18	5.94	56.12	80.84	8.08	88.92
**Mean annual IR** **(±** **SD)** **pre-PCV (2004–2008)**	**20.79 ** **(±** **7.86)**	**3.41** **(±** **3.89)**	**24.19** **(±** **6.85)**	**12.27** **(±** **1.53)**	**2.39** **(±** **0.62)**	**14.65** **(±** **2.02)**	**49.41** **(±** **2.89)**	**8.18** **(±** **2.63)**	**57.59** **(±** **3.4)**	**82.74** **(±** **4.8)**	**6.52** **(±** **2.23)**	**89.26** **(±** **2.76)**
2008/09	21.20	4.50	25.70	9.64	0.00	9.64	32.12	7.71	39.83	82.18	9.20	91.39
2009/10	10.62	5.62	16.24	6.87	3.12	10.00	29.99	5.62	35.61	31.38	11.53	42.91
2010/11	14.05	3.67	17.72	7.94	2.44	10.38	14.66	9.77	24.43	31.68	14.29	45.96
2011/12	16.21	8.41	24.62	1.80	1.80	3.00	1.20	17.41	18.61	7.86	21.78	29.64
2012/13	2.36	2.36	4.72	1.18	3.54	4.72	5.31	16.53	21.84	1.19	23.17	24.36
2013/14	5.23	7.55	12.78	2.91	5.23	8.13	2.91	14.53	17.43	2.34	32.78	35.12
2014/15	2.28	8.56	10.84	1.14	3.99	5.13	3.99	13.69	17.68	1.15	31.68	32.83
2015/16	1.12	6.73	7.85	0.56	3.37	3.93	2.24	19.07	21.31	1.13	25.45	26.58
2016/17	2.77	6.64	9.41	0.55	7.75	8.30	2.21	17.16	19.37	1.67	25.01	26.68
2017/18	0.55	8.21	8.76	1.64	4.38	6.02	0.00	14.78	14.78	1.10	29.08	30.18
2018/19	1.64	8.20	9.84	0.55	4.92	5.47	2.19	22.42	24.60	1.63	22.26	23.89
**Mean annual IR** **(±** **SD) ** **late-PCV (2016–2019)**	**1.65** **(±** **1.11)**	**7.69** **(±** **0.9)**	**9.34** **(±** **0.54)**	**0.92** **(±** **0.63)**	**5.68** **(±** **1.81)**	**6.59** **(±** **1.5)**	**1.65** **(±** **1.27)**	**17.94** **(±** **3.91)**	**19.59** **(±** **4.91)**	**1.46** **(±** **0.32)**	**25.44** **(±** **3.43)**	**26.91** **(±** **3.15)**
**IRR late vs pre-PCV** **(95% CIs)**	0.08 (0.02–0.26)	2.26 (0.81–6.26)	0.38 (0.21–0.68)	0.09 (0.02–0.39)	2.01 (0.63–6.42)	0.44 (0.22–0.89)	0.03 (0.01–0.11)	2.22 (1.14–4.29)	0.34 (0.23–0.50)	0.02 (0.01–0.06)	4.09 (2.00–8.35)	0.30 (0.22–0.42)

CI: confidence interval; IPD: invasive pneumococcal disease; IR: incidence rate; IRR: incidence rate ratio; NVT13: non-pneumococcal conjugated vaccine 13 serotypes; PCV13: pneumococcal conjugated vaccine; SD: standard deviation; VT13: pneumococcal conjugated vaccine 13 serotype.

The main variable suggesting the extent of the indirect protection provided by the 2+1 schedule is the impact on the PCV13 serotypes disease rates, especially in those not having received all doses of the vaccine. In the late-PCV13 period, VT13 IPD rates markedly declined in all age groups, compared to the pre-PCV period. In children < 4 months old, VT13 IPD rates declined by 92% (IRR: 0.08; 95% CI: 0.02–0.26), and in children 4–6 months old they declined by 91% (IRR: 0.09; 95% CI: 0.02–0.39). Similarly, IPD rates declined by 97% (IRR: 0.03; 95% CI: 0.01–0.11) in children 7–12 months old, and by 98% (IRR: 0.02; 95% CI: 0.01–0.06) in children 13–23 months old (Table, Figure).

**Figure:**
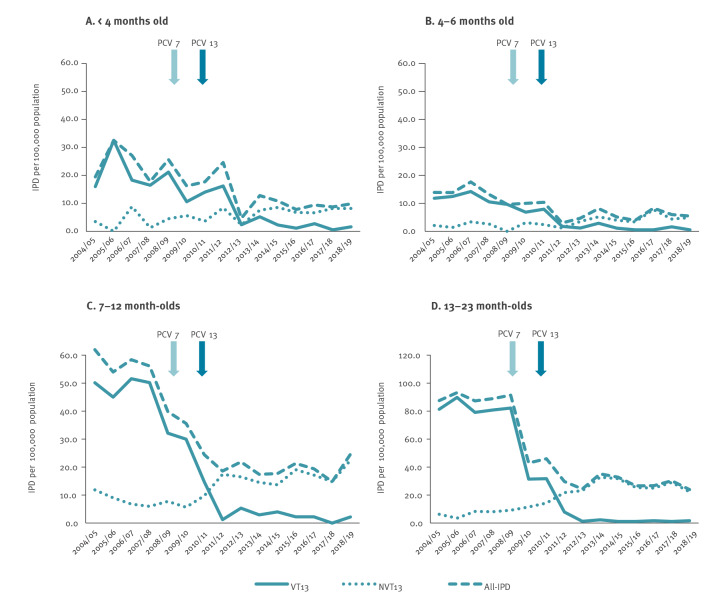
Invasive pneumococcal disease incidence rates in children aged < 4, 4–6, 7–12 and 13–23 months, Israel, 2004–2019 (n = 2,569 IPD episodes)

IPD: invasive pneumococcal disease; NVT13: non–pneumococcal conjugated vaccine 13 serotypes; PCV: pneumococcal conjugated vaccine; VT13: pneumococcal conjugated vaccine 13 serotypes.

### Dynamics of invasive pneumococcal disease caused by non-vaccine serotypes

In the pre-PCV period, the annual mean NVT13 IPD incidence rates per 100,000 ± SD were 3.4 ± 3.9 and 2.4 ± 0.6 in children < 4 and 4–6 months old, respectively, and 8.2 ± 2.6 and 6.5 ± 2.2 in children 7–12 and 13–23 months old, respectively (Table).

In the late-PCV13 period, NVT13 IPD rates appeared to increase 2–4-fold in all age groups, compared to the pre-PCV period rates. In children < 4 months old NVT13 IPD rates seemed to increase by 126% (IRR: 2.26; 95% CI: 0.81–6.26), and in children 4–6 months old they appeared to increase by 101% (IRR: 2.01; 95% CI: 0.63–6.42). The IPD rates increased by 122% (IRR: 2.22; 95% CI: 1.14–4.29) in children 7–12 months old, and by 309% (IRR: 4.09; 95% CI: 2.00–8.35) in children 13–23 months old.

### Dynamics of overall invasive pneumococcal disease

It is important to assess whether the marked impact observed on PCV13 serotype IPD rates in all age groups resulted also in impact on the overall IPD rates in all age groups. In the pre-PCV period (July 2004 through June 2008), overall IPD incidence rates per 100,000 individuals ± SD were stable and were 24.2 ± 6.9 and 14.7 ± 2.0 in children < 4 and 4–6 months old, respectively, and 57.6 ± 3.4 and 89.3 ± 2.8 in children 7–12 and 13–23 months old, respectively (Table).

In the late-PCV13 period (July 2016 through June 2019), overall IPD rates significantly declined in all age groups, when compared to the pre-PCV period rates. In children < 4 months old IPD rates declined by 62% (IRR: 0.38; 95% CI: 0.21–0.68), and in children 4–6 months old they declined by 56% (IRR: 0.44; 95% CI: 0.22–0.89). Similarly, IPD rates declined by 66% (IRR: 0.34; 95% CI: 0.23–0.50) in children 7–12 months old, and by 70% (IRR: 0.30; 95% CI: 0.22–0.42) in children 13–23 months old.

Overall, no difference between ethnic groups was observed regarding IRRs throughout the periods (data not shown).

## Discussion

PCV13 implementation in Israel with the 2+1 schedule resulted in indirect protection of young infants, including infants < 4 months old who did not receive more than one PCV dose. In all studied age groups < 13 months of age, VT13 rates declined by > 90%, and overall IPD rates declined by 56–66%, when the late-PCV period was compared to the pre-PCV period. These reductions were in the same order of magnitude as those for 13–23-month-old children (70%) who were fully vaccinated, including the booster dose. The impact on all-IPD was substantial, despite the trend of increasing NVT13 IPD rates.

The impact of PCV13 on IPD incidence in infants < 4 months old is evidence for indirect protection, achieved through prevention of new acquisition of VT13 nasopharyngeal carriage in fully vaccinated children, which resulted in reduced transmission of VT13 in the entire population [5]. In a prospective randomised study comparing immunogenicity and pneumococcal nasopharyngeal acquisition of a 2+1 (4, 6, and 12 months) with a 3+1 (2, 4, 6, and 12 months) PCV7 schedule, we showed that the primary two-dose infant series resulted in lower serotype-specific anticapsular IgG concentrations and higher nasopharyngeal acquisition of PCV7 serotypes than the primary infant three-dose schedule, suggesting that during the pre-booster interval, infants receiving two PCV7 doses were more vulnerable to pneumococcal disease than those who had received three doses [17]. However, after booster administration, vaccine-serotype acquisition rates were similar among the two groups, suggesting equal post-booster potential for indirect protection.

Indirect protection has been mainly described for adults [16]. Our findings allow real appreciation of the full impact of PCV13 on additional age groups. This is of importance for public health policy makers, since young infants are, on one hand, at high risk for pneumococcal disease and, on the other, only partially vaccinated at best. An important insight is that the vaccination coverage of the booster dose is a crucial factor in the success of a 2+1 vaccination schedule. Since booster vaccination rates are high in Israel, our findings are not necessarily extrapolatable to countries with markedly lower booster administration rates.

Another important finding in our study is that the 2+1 schedule in Israel was highly protective for those who had not yet completed the full vaccination schedule, suggesting that the vaccine impact provided by the 2+1 schedule is similar to that by the 3+1 schedule. The 2+1 schedule is used in practically all European countries, of which the majority use PCV13, and most administer the first two doses at 2- and 4- or 3- and 5-months schedules, and the third dose early in the second year of life, similar to the Israeli 2+1 schedule [18]. Studies are needed to confirm if the 1+1 schedule, recently introduced in the United Kingdom, provides indirect protection at a level similar to the 2+1 schedule.

As expected, we observed that IPD caused by NVT13 serotypes appeared to increase across all age groups resulting in an offset of the overall decline in IPD. However, despite the increase in NVT13 serotypes IPD rates, the all-IPD rates declined markedly by 62%, 56%, 66%, and 70% among the < 4-month-, 4–6-month-, 7–12-month-, 13–23-month-old children. The Israeli Advisory Committee on Infectious Diseases and Immunisation has recently recommended to replace PCV13 by the recently developed 20-valent PCV (PCV20). The seven additional serotypes in PCV20 beyond those in PCV13 serotypes (8, 10A, 11A, 12F, 15B/C, 22F and 33F), grouped constitute 52% of the remaining IPD in children < 2 years in Israel [19], suggesting further potential reduction in IPD. However, the effectiveness of PCV20 against the new serotypes it targets, and the resulting non-PCV20 serotype IPD rate increase are yet unknown, as well as the impact of the PCV20 on nasopharyngeal carriage of the additional PCV20 serotypes.

The main strengths of the study include the prospective long duration of surveillance, starting several years in the pre-PCV era and continuing for 11 years following PCV implementation. Additionally, this is a nationwide study with large numbers of cases, enabling capture of practically all culture proven IPD, and thus reassuring that the study findings are well grounded.

We also acknowledge several limitations, including lack of data regarding non-culture-proven IPD episodes (e.g. PCR proven IPD), and the usage of only one regimen (2+1) in Israel throughout the study, and therefore not assessing other possible schedules (e.g. 1+1). To define IPD we used only positive blood and CSF cultures, and excluded PCR, antigen testing Gram stain or clinical diagnosis and some sources such as pleural, synovial and ascitic fluids. This was necessary to obtain a stable methodology over the years (since some of the sensitive diagnostic methods were not routinely used in the first years of the study) and across centres. This was potentially associated with somewhat lower incidence estimates, but on the other hand provided stability and universality which permitted a more adequate evaluation of the incidence dynamics. Furthermore, delays in vaccination could lead to an overestimation of protection in the 7–12 months group. However, such delays could, at the same time, lead to underestimation of the protection in the 4–6 months group. While data on main diagnoses, outcome (mortality), and hospitalisation duration were collected during the study, their analysis was not presented, since the study focused on prevention of IPD in general rather than more specific protection against severe disease.

## Conclusion 

PCV13 implementation in Israel resulted in indirect protection for young infants, including infants < 4 months who received only ≤ 1 PCV dose. Additionally, the 2+1 schedule used in Israel seems adequate, as the impact on infants 7–12-month-old (receiving only 2 infant doses) resulted in close to complete elimination of VT13 IPD, similar to that in children 12–23-month-old (receiving all 3 vaccine doses including the booster dose).
